# Nanotopography of Polystyrene/Poly(methyl methacrylate) for the Promotion of Patient Specific Von Willebrand Factor Entrapment and Platelet Adhesion in a Whole Blood Microfluidic Assay

**DOI:** 10.3390/polym15061580

**Published:** 2023-03-22

**Authors:** Joanna Ward, Eimear Dunne, Ingmar Schoen, Adrian R. Boyd, Dermot Kenny, Brian J. Meenan

**Affiliations:** 1 Nanotechnology and Integrated Bioengineering Centre (NIBEC), School of Engineering, Ulster University, York Street, Belfast BT15 1AP, UK; 2 Irish Centre for Vascular Biology, Royal College of Surgeons in Ireland, 123 St Stephen’s Green, D02 YN77 Dublin, Ireland

**Keywords:** surface nanotopography, dynamic platelet function assay, platelet tracking algorithm, polymer demixing, spin coating

## Abstract

Platelet function testing is essential for the diagnosis of patients with bleeding disorders. Specifically, there is a need for a whole blood assay that is capable of analysing platelet behaviour in contact with a patient-specific autologous von Willebrand factor (vWF), under physiologically relevant conditions. The creation of surface topography capable of entrapping and uncoiling vWF for the support of subsequent platelet adhesion within the same blood sample offers a potential basis for such an assay. In this study, spin coating of polystyrene/poly (methyl methacrylate) (PS/PMMA) demixed solutions onto glass substrates in air has been used to attain surfaces with well-defined topographical features. The effect of augmenting the PS/PMMA solution with uniform 50 µm PS microspheres that can moderate the demixing process on the resultant surface features has also been investigated. The topographical features created here by spin coating under ambient air pressure conditions, rather than in nitrogen, which previous work reports, produces substrate surfaces with the ability to entrap vWF from flowing blood and facilitate platelet adhesion. The direct optical visualisation of fluorescently-labelled platelets indicates that topography resulting from inclusion of PS microspheres in the PS/PMMA spin coating solution increases the total number of platelets that adhere to the substrate surface over the period of the microfluidic assay. However, a detailed analysis of the adhesion rate, mean translocating velocity, mean translocation distance, and fraction of the stably adhered platelets measured during blood flow under arterial equivalent mechanical shear conditions indicates no significant difference for topographies created with or without inclusion of the PS microspheres.

## 1. Introduction

Von Willebrand’s disease (vWD) is the most common bleeding disorder, with approximately 1% of people affected globally [1]. It is characterised by a deficiency in the blood protein von Willebrand factor (vWF) [2]. Types 1, 2, and 3 vWD are distinguished by lower-than-normal concentrations of vWF [3], abnormal defects within vWF multimer structure, or a total absence, respectively [4,5]. A clinical diagnosis of vWD is made using multiple clinical criteria [4,6], including a personal history of abnormal bleeding, family history of bleeding disorders, and reduced levels of vWF [7]. However, these factors may not all be present, making a clear diagnosis difficult, with many patients only being identified following an abnormal bleeding event. Diagnostic testing, such as the vWF:Antigen assay, which quantifies the concentration of vWF in circulating blood, is widely used to diagnose vWD and to determine its specific sub-type [8]. To confirm type 2 vWD, platelet function assays (PFAs) such as the Ristocetin cofactor are used to assess platelet:vWF interactions as a function of vWF availability [9]. The assessment of vWF in conjunction with platelet interaction is essential in understanding vWF activity in both healthy individuals and those with vWD. A significant limitation of the current gold standard assay techniques is that they are all carried out under static conditions, meaning they are not physiologically representative. Moreover, many of them do not directly measure the interaction of a specific patients’ platelets with the same individual’s vWF.

The role of nanotechnology and nanomaterials is increasing across a number of applications [10,11]. The fabrication of micro- and nanotopographies can facilitate blood-sensing technologies [12]. The use of microfluidic flow chambers as the basis of dynamic platelet assays has been in development for some time [13]. The most modern version of such devices facilitates dynamic blood flow at shear rates equivalent to arterial blood flow to better mimic the in vivo conditions [14,15]. In addition, the microfluidic chambers only require small volumes of blood for diagnosis and provide the opportunity for real-time monitoring, in accordance with the requirements of the International Society of Thrombosis and Haemostasis standards [16].

Previous studies have reported the use of the coatings of adhesive proteins, such as collagen and fibrinogen [17,18], which are adhered to the surface of flow channels in microfluidic devices to study platelet response thereon. In a bid to facilitate the assessment of the platelets directly interacting with vWF, a dynamic platelet function assay (DPFA) has been developed [19,20,21,22,23]. This system is based on a disposable parallel-plate flow chamber with endogenous vWF immobilised onto the bottom of the flow channel, which acts to identify platelet adhesion and activation when in contact with fluorescently-labelled platelets in whole blood. Detailed information about the dynamic platelet behaviour is obtained from single platelet tracking data derived from static images from video footage acquired during flow, as well as from the computational modelling of detected platelets [24,25,26,27].

In its normal configuration, the DPFA lacks specificity, with respect to a specific patient’s vWF, due to the use of the endogenous vWF. The replacement of this externally-derived protein with a surface topography that can entrap autologous vWF from flowing whole blood, which can then support subsequent platelet adhesion from whole blood under arterial shear, has been suggested as a means of addressing this limitation of the DPFA [28,29]. Previously published work by the authors has reported a positive platelet response to surfaces created by spin coating a solution of polystyrene(PS)/poly(methyl methacrylate) (PMMA) onto the glass substrates used as the flow channel in the DPFA device [30]. The ability of these surfaces to entrap vWF and capture platelets is attributed to the nanoscale topography that results from the spin coating of the demixed polymer solution. The addition of small amounts of 50 µm PS microspheres to the PS/PMMA solution has been used to enhance the regularity and reproducibility of the associated DPFA flow plate substrate. This, and the related previous work, has used a nitrogen environment during the spin coating process to aid the rapid evaporation of the chloroform solvent.

In this study, a range of polymer demixed surfaces have been prepared from PMMA/PS and PMMA/PS + PS microsphere solutions in the air under ambient conditions and incorporated within the flow chamber of the DPFA with the resulting surfaces characterised both physically and chemically. Haematological analysis was carried out using fluorescently-labelled platelets in whole blood, from a single donor, perfused over the flow channel at a rate of 1500 s^−1^. Video rate image capture was used to undertake real-time dynamic tracking of platelet kinetics under standard DPFA conditions. As in previous studies, the total number of platelets that adhere to the substrate surface over the period of the microfluidic assay have been measured. In addition, a bespoke computer algorithm, developed to determine the kinetic behaviour of platelets on endogenous vWF-coated substrates, has, for the first time, been adapted for the measurement of platelet behaviour on the polymer demixed synthetic surfaces of interest here.

## 2. Materials and Methods

### 2.1. Sample Preparation

Polystyrene (PS, Mw = 290,000 amu) and poly (methyl methacrylate) (PMMA, Mw = 350,000 amu) (Sigma Aldrich, Irvine, UK) were used to create a 25%PS/75%PMMA polymer blend, from which a 3% casting solution was created in chloroform (119.38 g/mol, Sigma Aldrich, UK). This polymer blend solution acts as the base spin coating system, which was then augmented at a 1:4 (v/v) ratio with uniform 50 µm PS microspheres (ThermoFisher, Loughborough, UK). The 25PS75PMMA baseline polymer blend has been associated with producing a topography that can produce a positive platelet interaction [28,29]. More specifically, the influence of incorporating uniform microspheres in varying amounts has been studied previously under standard nitrogen purge, with spin coating conditions with 750 µspheres/mL (1:4 ratio) yielding the most appropriate surface topography change [29]; thus, 25PS75PMMA and 25PS75PMMA_750 where investigated. The method for creating the microsphere augmented solutions is described in detail elsewhere [29]. In brief, a 5 mL aliquot of aqueous microspheres was oven-dried and washed into 20 mL of the 3% 25/75 PS/PMMA blend solution, denoted 25PS75PMMA_750. Glass coverslips (24 × 50 mm, Marienfeld-Superior, Lauda-Königshofen, Germany), onto which the polymer demixed solutions were spin coated, were cleaned with 99% isopropyl alcohol (Sigma Aldrich, Irvine, UK) using an ultrasonic bath (Ultrawave Ltd., Cardiff, UK). Slides were sonicated for 30 min before being rinsed with deionised water and placed into an oven operating at 70 °C to dry.

### 2.2. Spin Coating

Rigorously cleaned glass coverslips were placed on a vacuum chuck of a VTC-100 compact spin coater (MTI Corporation, Richmond, VA, USA). The polymer casting solution was dispensed onto the glass slide until the surface was saturated, and the spin programme was then initiated. The spin profile encompassed an initial 2-min period, whereby speed was ramped up to 2000 RPM at a steady rate, before a 2-min ramp-down period. This instrument operates in air, under atmosphere conditions, i.e., there is no nitrogen purge present, and coated substrates are denoted by the term ‘air-spun’ substrates. Coated coverslips were removed from the chuck and stored in a clean, dry environment until required.

### 2.3. Surface Characterisation

Physical characterisation of the surfaces of air-spun coated substrates was carried out using atomic force microscopy (AFM) to determine the nature and scale of the resulting topography. Scans were acquired on a Veeco Digital Instrumentation Dimensions 3100 (Bruker Axs, Coventry, UK) at a scan rate of 0.5 Hz across areas of 40 × 40 µm. Data were collected in the form of 3D visual plots and surface roughness parameters—Ra (arithmetic mean roughness) and Rq (root mean square roughness)—and line profiles generated from the midpoint, perpendicular to the xy scan direction, were also recorded (n = 5).

X-ray photoelectron spectroscopy (XPS) was used for chemical characterisation of the surfaces using a Kratos Axis Ultra Delay Line Detector spectrometer system (Kratos Analytical Ltd., Manchester, UK) equipped with Kα X-rays operating at an incident energy of 1486.6 eV. Wide energy survey scans (WESS) were collected at a pass energy of 160 eV and high-resolution spectra at 20 eV. Charge neutralisation was applied due to the insulating nature of the polymer thin films. This was applied via an electron gun operating at 1.95 A with a charge balance of 3.3 V. Post-acquisition, data were analysed using CasaXPS (version 2.3.19), where adventitious carbon was calibrated to 285.0 eV before peak fitting was completed for both oxygen and carbon spectra.

### 2.4. Haematological Assessment

Ethical approval for the study was obtained from the Research Ethics Committee of the Royal College of Surgeons in Ireland (RCSI REC approval number 12/08). All blood samples were collected in accordance with the Declaration of Helsinki [31]. Blood was collected from a single healthy donor with normal vWF levels and normal platelet function. Whole blood samples were stained with 1 µM 3-3′-dihexyloxacarbocyanine iodide (DIOC6); a fluorescent dye that can be excited at 488 nm. The donor’s age and gender were recorded, and platelet indices were measured at the time of donation using a Sysmex KX21N (Sysmex, Kobe, Japan) haematology analyser. Table 1 shows a representative blood profile for the healthy donor.

### 2.5. Dynamic Platelet Function Assay

Dynamic platelet function tests were carried out within a DPFA flow assembly, described in detail elsewhere with relevant schematics [19,21,23,29]. In brief, glass substrates with air-spun PS/PMMA and PS/PMMA_750 coatings were attached to a PMMA top plate via a pressure-sensitive adhesive (PSA) tape. The top plate is fitted with inlet and outlet ports at either end of a defined groove etched into the underside of the polymer top plate, such that the substrate surface seals and completes the flow channel. The microfluidic device was designed in earlier research works to yield an aspect ratio of 0.025 (1:40) and a cross-sectional area of 0.1 mm^2^ [23], which yields more efficiency in reaching arterial shear. The test assembly is then mounted onto the stage of a Zeiss Axiovert-200 epifluorescence inverted microscope (Zeiss, Wexford, Ireland). A sample of blood labelled with the DIOC6 fluorescent dye was perfused across the flow channel via the inlet/outlet ports at a flow rate of 75 µL/min, equivalent to an arterial shear of 1500 s^−1^. Optical images of the flow path were collected using an air-cooled (−80 °C) digital iXON EM+ CCD camera (Andor Technology, Belfast, UK) operating in a video capture mode using MetaMorph (Molecular Devices Ltd., Wokingham, UK) software, whilst illuminating the area with an Osram 103-W light source equipped with a fluorescein iosthiocyanate (FITC) filter set (Chroma Technology Corp, Rockingham, VT, USA).

On operator observation of the first platelets sticking to the surface, data collection was initiated, recording images at 19 frames per second (fps) for 1000 frames, followed by time lapse image acquisition of 120 frames at 0.5 fps. In all instances, the final frame from the time lapse image stack (120/120) was used to quantify % surface coverage using the Volocity™ software package (Perkin-Elmer, Buckinghamshire, UK). Platelets were masked using a “find objects by % intensity” thresholding tool using upper and lower limits of 100 and 85, respectively. Total area highlighted was then measured in pixels, with respect to the full image pixel number, and the percentage coverage was calculated.

### 2.6. Dynamic Platelet Analysis Algorithm

Datasets from the initial 1000-frame stack were analysed using an established algorithm [24]. In short, platelets were detected by a combination of intensity thresholding and template matching, localised with sub-pixel accuracy, and tracked through subsequent frames. Minor adaptions were made to account for the lower frame rate of 19 fps (previously 30 fps) and the higher background signal from the PMMA substrate. As before, platelet adhesion rate (×10^3^/s), mean translocating velocity (µm/s), mean translocation distance (µm), and fraction of stably adhered platelets were measured. Table 2 describes the biological importance of these measures.

## 3. Results

### 3.1. Characterisation of Air-Spun PS/PMMA and PS/PMMA_750 Thin Films

**Figure 1:**
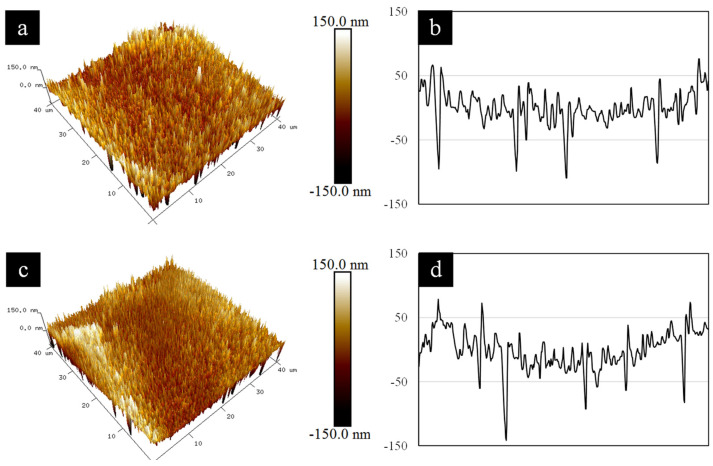
Pseudo−colour 40 μm × 40 μm 3D AFM plots for (**a**) 25PS75PMMA and (**c**) 25PS75PMMA_750 surfaces, (colour scale range ± 150 nm) with corresponding line profiles (**b**,**d**) generated from xy midpoint.

Coatings of PS/PMMA and PS/PMMA_750 spin coated, in air, onto glass substrates were characterised by atomic force microscopy (AFM) and x-ray photoelectron spectroscopy (XPS) before being subject to haematological assessment. AFM analysis provides the physical characterisation of the surface topographical features, with Figure 1 showing representative 3D plots and corresponding line profiles for 25PS75PMMA (a, b) and 25PS75PMMA_750 (c, d), respectively.

No significant differences between the data for the 25PS75PMMA and 25PS75PMMA_750 surfaces are evident from Figure 1, with both substrates seen to have sharp features ranging from −145 nm to 75 nm. The 25PS75PMMA substrate has an average Ra = 22.30 ± 0.60 nm and Rq = 31.1 ± 2.50 nm, whilst the 25PS75PMMA_750 surface shows values of Ra = 25.20 ± 3.20 nm and Rq = 34.90 ± 2.70 nm. Comparison of the mean surface roughness showed no statistically significant difference (*p* > 0.05), whilst the root mean square roughness was significantly different between substrates.

**Figure 2:**
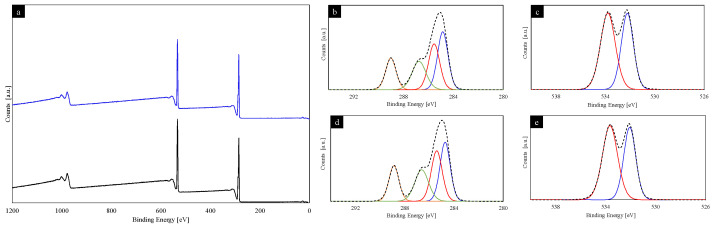
XPS spectral comparison for (**a**) wide energy survey scans of 25PS75PMMA (black) and 25PS75PMMA_750 (blue) substrates with related deconvoluted high energy plots for C 1s of (**b**,**d**) showing blue, red, green, orange and dotted lines relating to C-H hydrocarbon, C-C-O beta-shifted carbon, C-O methoxy group and O-C=O carbonyl bond and spectral envelope, respectively and O 1s (**c**,**e**) showing blue, red and dotted lines corresponding to O=C carbonyl, O-C methoxy group oxygens and the oxygen spectral envelope, for 25PS75PMMA and 25PS75PMMA_750 substrates, respectively.

As shown in Figure 2, XPS analysis revealed that both substrates present a PMMA-dominant surface chemistry with only PMMA-characteristic environments present within the high resolution carbon (C 1s) and oxygen (O 1s) spectra recorded [32].

Quantitative analysis of the deconvoluted C 1s and O 1s contributions to the spectral envelopes are presented in Table 3 as average % atomic concentration (n = 3) ± standard deviation (SD).

### 3.2. Haematological Assessment

The 25PS75PMMA and 25PS75PMMA_750 substrates were subjected to haematological analysis using the DPFA protocol. Data were recorded by way of obtaining fluorescent images from the observation of the adherence of the first platelet to the surface across a total of 5 min, comprising an initial 1000-frame stack, followed by time lapse of 120 frames at lower acquisition rate, with the final frame (120/120) used for direct visual comparison of the surface coverage of the platelets.

**Figure 3:**
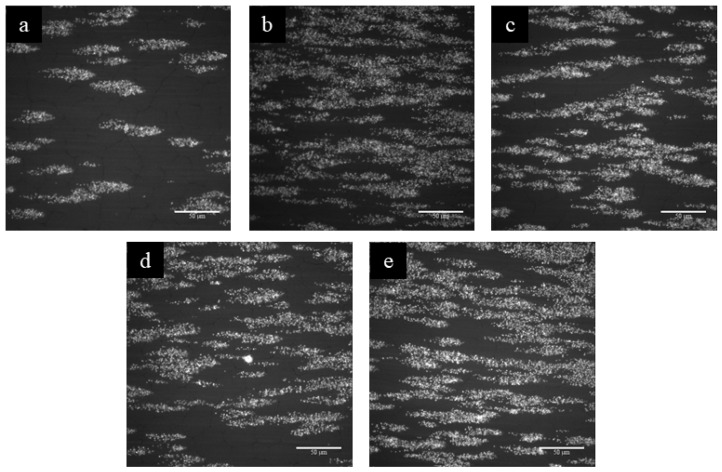
Fluorescence micrographs of DIOC6 labelled whole blood platelets detected in frame 120/120 for 5 replicates (**a**–**e**) of the air-spun 25PS75PMMA surface (Scale bar = 50 µm).

Figure 3 shows the fluorescent micrographs of five replicates of the air-spun 25PS75PMMA substrate surfaces. There appears to be some variation across the five replicates, in particular, with sample (a) showing a considerable reduction in the number of platelets adhered to the surface. Quantitative data for the percentage surface coverage is provided in Table 4 and shows an average (n = 5) surface coverage of 17.30 ± 6.8% within the field of view.

**Figure 4:**
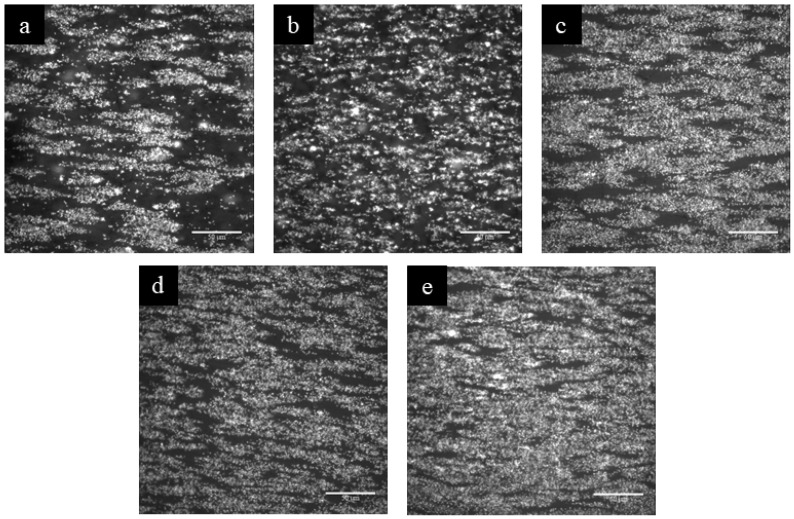
Fluorescence micrographs of DIOC6 labelled whole blood platelets in frame 120/120 for each of 5 replicates (**a**–**e**) of the air-spun-coated 25PS75PMMA_750 surface (Scale bar = 50 µm).

Fluorescent images for the five replicates of the equivalent air-spun 25PS75PMMA_750 substrates analysed within the DPFA assembly are provided in Figure 4.

Platelet adhesion appears to be significantly higher for this surface, with platelets covering a large proportion of the field of view. Surface coverage values are again shown in Table 4 and indicate an average (n = 5) of 40.20 ± 13.30% surface coverage within the field of view. Within the regions covered by platelets, there are significantly ‘brighter’, more densely fluorescent areas that indicate the formation of 3D thrombi building within the microfluidic channel.

### 3.3. Dynamic Analysis of Platelet Behaviours

The dynamic behaviour of platelets across the 25PS75PMMA and 25PS75PMMA_750 surfaces was assessed from the initial 1000-frame image stack to ensure that only platelet-surface interactions were analysed, in order to negate (as far as possible) platelet-platelet and thrombotic activities. 

Table 5 provides the dynamic platelet translocation parameters (average ± SD) resulting from both air-spun polymer demixed surfaces. The relevant platelet indices measured at the time of blood collection for each sample type are also provided.

Whilst both the mean translocating distance and mean translocating velocity appear to slightly decrease with the addition of 50 µm spheres, both the total number of tracks and fraction of stably adhered platelets appears to increase. However, since the sample-to-sample variability was high (see Table 5), no statistically significant difference (*p* > 0.05) was evident from the number of runs performed (n = 5). These data confirm that, whilst there are clear differences on visual inspection of the images (120/120) captured, there is no statistically significant difference in the dynamic behaviour of platelets on each type of substrate topography.

## 4. Discussion

The surfaces created by spin coating of 25PS75PMMA and 25PS75PMMA_750 polymer demixed solutions in air under ambient conditions have been analysed with regard to the respective physical and chemical properties. Their respective ability to entrap vWF and bind platelets from flowing whole blood has been determined using a dynamic platelet function assay.

The AFM results show that the topography of the 25PS75PMMA surface consists of sharply defined features (FWHM 500 nm to 1 µm) ranging from −100 to +75 nm in height. This is a significantly different topography than that created by spin coating the same PS/PMMA blend under nitrogen purge conditions, where it was found that the features were much more rounded and globular [29]. The air-spun 25PS75PMMA_750 substrates show the same sharp, closely packed protrusions (FWHM < 500 µm), spanning from −145 to +140 nm. The creation of sharper features in both samples is believed to result from the slightly faster evaporation of the chloroform solvent in air, compared to the conditions that prevail during a pressure nitrogen purge [34,35]. This premature evaporation of the chloroform results in sharp features being present across the substrate, irrespective of polymer blend, showing no differences, in relation to surface features, on a visual basis.

XPS analysis confirms that the uppermost surface of both the air-spun 25PS75PMMA and 25PS75PMMA_750 substrates is that of PMMA only [32]. Small differences within the deconvoluted C1s spectra components are observed, however, and it is likely that this is attributable to slight differences in the orientation of the polymer chains as a result of the spin coating process [33]. These findings are consistent with previous studies by the authors of the chemical properties of spin coating of PS/PMMA blends in nitrogen, which leads to the component with the higher surface energy (PMMA) being segregated at the uppermost surface [28,29,36]. This suggests that, notwithstanding the volatility of the chloroform solvent, which results in evaporation before the system has reached thermodynamic equilibrium [37], the segregation of PMMA to the uppermost surface still occurs. However, it is noted that this finding is, again, contradictory to the results from other reports in the existing literature [38].

The haematological response to the 25PS75PMMA and 25PS75PMMA_750 substrates was assessed by way of a DPFA test protocol, which has been reported previously [19,21]. Previous work shows that surface-bound and uncoiled endogenous vWF is required to enable platelet adhesion and aggregation in vivo [28,29]. Therefore, synthetic polymer topographies of the type created in this work must facilitate the initial vWF binding and then provide for its subsequent uncoiling from whole blood under arterial shear, in order to expose binding sites that are then available for subsequent platelet interactions. The results obtained here indicate that both 25PS75PMMA and 25PS75PMMA_750 substrates can elicit such a dynamic platelet response and, therefore, by association, support vWF entrapment and uncoiling. The addition of PS microspheres (25PS75PMMA_750) to the standard polymer demixed spin coating solution results in more than double (40.20 ± 13.30%) the number of surface adhered platelets at the end of assay, compared to that on the base (25PS75PMMA) surface (17.30 ± 6.80%). However, the standard deviation for the % surface coverage values across five samples of both substrate types is seen to be relatively high, which presents a challenge for the potential use of a PS/PMMA polymer demixed spin coated surface as a synthetic sensor substrate regarding reproducibility. It is noted that the deliberate use of blood from a single healthy donor and the consistency of the platelet indices for each set of DPFA measurements negates this as a significant source of variance in the data. Visual inspection of the fluorescent micrographs alone suggests that 25PS75PMMA_750 substrates outperform the 25PS75PMMA surfaces, with respect to supporting platelet adhesion. However, the statistical analysis of key dynamic behaviour parameters of translocating platelets, i.e., adhesion rate, mean translocating velocity, mean translocation distance, and fraction of stably adhered platelets reveals no statistically significant difference in platelet behaviour on either type of air-spun polymer surface. This result is deemed to be due to the large sample-to-sample variance observed in the fluorescent micrographs.

Nanotopographies created from 25PS75PMMA and 25PS75PMMA_750 blends present novel substrates that facilitate a high level of platelet adhesion and, therefore, vWF binding. This is a significant improvement in the field of platelet function assay testing, as it provides an opportunity for patient-specific platelet and protein (vWF) assessment from a single, whole blood sample.

## 5. Conclusions

Polymer demixed thin films fabricated by spin coating PMMA/PS in chloroform solutions with and without the addition of PS microspheres onto glass substrate have surface topographies capable of entrapping vWF from whole blood flowing at a rate of 75 µL/min, equivalent to an arterial shear of 1500 s^−1^, which then directly promotes the subsequent platelet interactions thereon. Images captured at the end of flow experiments show a substantial increase in the surface coverage of platelets on the 25PS75PMMA_750 substrate. The assessment of associated platelet dynamics on both types of substrates during the time period of the assay using a model adapted from previous studies with immobilised vWF surfaces indicates no significant difference in translocation behaviour, in terms of adhesion, velocity, or distance travelled. Whilst 25PS75PMMA substrates show a lesser number of platelets covering the surface at the end of analysis, those platelets present successfully interact with the respective topographies. The findings from this study significantly extend our knowledge of the potential use of air-spun polymer demixed substrates for dynamic platelet assay that offer patient-specific protein-platelet screening from a single blood sample. Whilst the work produced within this study addresses the research question asked, it is noted that there are associated limitations. More specifically, further research is needed to understand the accuracy of this microfluidic assay against commercially existing assays to validate its application, and the sample set needs to be widened to include different blood groups. Future works to develop this research, focusing on a collective of blood donors, are required, in order to investigate the different blood types and health conditions (healthy/von Willebrand disease patients) and gain an insight on the robustness of this new synthetic surface as a sensor for the diagnosis of vWD and associated diseases that effect vWF-platelet interactions.

## Figures and Tables

**Table 1:** Blood profile for healthy donor showing platelet indices relevant to platelet activation.

Name	Donor 1	Haematocrit (HCT)	38.9%
Age	24	Platelet Count (PLT)	260 × 10^3^/µL
Blood Type	O	Mean Platelet Volume (MPV)	9.4 fL
Gender	Female	Platelet-Large Cell Ratio (P-LCR)	17.6%

**Table 2:** Description of platelet parameters measured and their biological relevance.

Parameter	Biological Relevance
Platelet adhesion rate (×10^3^/s)	Rate of change in platelet adhesion to the surface, which is calculated by plotting a regression line against the cumulative number of platelets. A higher platelet adhesion rate indicates that platelets are ‘stickier’ or more likely to form a thrombus.
Mean translocating velocity (µm/s)	Mean velocity is an indication of how fast platelets are translocating. A reduction in platelet translocation speed may indicate increased signalling within the platelet, causing faster activation of GP IIb/IIIa, which slows down platelets and leads to stable adhesion.
Mean translocating distance (µm)	Average distance travelled by translocating platelets. A decrease in this value can indicate increased signalling within the platelet, which leads to faster activation of GP IIb/IIIa, reducing the distance a platelet travels before stably adhering to the surface.
Fraction of stably adhered platelets	Total number of platelets that are stably adhered to the surface as a fraction of all detected platelet tracks. This is defined as platelets moving less than 1.5× their own radius over the whole duration of the movie. In biological terms, this refers to the number of platelets that initially interact with autologous vWF and remain adhered to the surface.

**Table 3:** XPS derived average (n = 3) % atomic concentration of the C 1s and O 1s deconvoluted components for 25PS75PMMA and 25PS75PMMA_750 substrate surface.

Substrate	C 1s				O 1s	
	C-H	C-C	C-O	C=O	O=C	O-C
25PS75 PMMA	33.48 ± 1.17	20.97 ± 0.67	28.27 ± 1.62	17.28 ± 0.37	45.22 ± 0.68	54.78 ± 0.68
25PS75 PMMA_ 750	34.24 ± 3.42	22.35 ± 0.77	25.53 ± 3.04	17.88 ± 0.47	44.20 ± 0.70	55.80 ± 0.70

The data presented here indicates that the surface chemistry of air-spun-coated PS/PMMA does not appear to change significantly with the introduction of PS microspheres, which supports the findings of previous studies [33].

**Table 4:** Average % surface coverage values (n = 5) ± standard deviation (SD) taken from frame 120/120 of fluorescently-labelled platelets on 25PS75PMMA and 25PS75PMMA_750 substrates after DPFA testing.

Substrate	Sample	Surface Coverage (%)	Average (%) ± SD
25PS75PMMA	1	8.00	17.30 ± 6.80
	2	14.10	
	3	20.00	
	4	18.00	
	5	26.20	
25PS75PMMA_750	1	27.70	40.20 ± 13.30
	2	26.90	
	3	48.40	
	4	39.80	
	5	57.90	

**Table 5:** Dynamic platelet parameters derived from the autologous platelet function algorithm analysis (n = 5 ± SD) and the associated relevant platelet indices from haematological analysis of the healthy donor blood.

Dynamic Parameters	25PS75PMMA	25PS75PMMA_750	*p*-Value
Total number of tracks per FOV	168 ± 44	474 ± 331	0.07
Fraction of stably adhered platelets	0.75 ± 0.03	0.78 ± 0.05	0.24
Mean translocating distance (μm)	2.22 ± 0.31	2.13 ± 0.38	0.68
Mean translocating velocity (μm/s)	1.44 ± 1.06	1.21 ± 0.64	0.69
Platelet adhesion rate (×10^3^ s^−1^)	0.69 ± 0.15	1.65 ± 1.27	0.14
Platelet detachment rate (s^−1^)	1.30 ± 1.89	2.20 ± 1.33	0.41
Fraction platelets sticking	0.47 ± 0.18	0.49 ± 0.09	0.79
Platelet Indices *			
Platelet count (×10^3^/μL)	224	260	
Haematocrit (%)	31.2	38.9	
vWF (IU/mL)	47.6	47.6	

* Single values for indices are provided as tests were carried out with a single donor sample.

## Data Availability

Not applicable.
